# Quantitative analysis of macrophage inhibitory cytokine-1 (MIC-1) gene expression in human prostatic tissues

**DOI:** 10.1038/sj.bjc.6600869

**Published:** 2003-04-01

**Authors:** T Nakamura, A Scorilas, C Stephan, G M Yousef, G Kristiansen, K Jung, E P Diamandis

**Affiliations:** 1 Department of Pathology and Laboratory Medicine, Mount Sinai Hospital, Toronto, Ontario, Canada M5G 1X5; 2 National Center of Scientific Research ‘Demokritos’, IPC, 153 10 Athens, Greece; 3 Department of Urology, University Hospital Charité, Humboldt University, D-10098 Berlin, Germany; 4 Department of Laboratory Medicine and Pathobiology, University of Toronto, Toronto, Ontario, Canada M5G 1L5; 5 Department of Pathology, University Hospital Charité, Humboldt University D-10098 Berlin, Germany

**Keywords:** macrophage inhibitory factor-1, prostate cancer, quantitative RT–PCR

## Abstract

Macrophage inhibitory cytokine-1 (MIC-1) gene is a member of transforming growth factor-*β* superfamily and was reported to be highly overexpressed in human prostate cancer using microarray technology. The aim of this study was to evaluate the quantitative expression of MIC-1 in malignant and benign prostate tissues and to associate expression levels with clinicopathological parameters of prostate cancer. Matched (paired) prostatic tissue samples from the cancerous and noncancerous parts of the same prostates were obtained from 66 patients who underwent radical prostatectomy. Quantitative RT–PCR was performed using SYBR Green I on the Roche LightCycler™ system. Macrophage inhibitory cytokine-1 gene overexpression in cancerous tissues was observed in 88% of cases, compared to noncancerous tissues (*P*<0.001). The expression level of MIC-1 in cancerous tissues was significantly higher than in noncancerous tissue (*P*<0.001). Higher expression of MIC-1 gene was significantly associated with higher Gleason score (*P*=0.004). The expression of the MIC-1 gene in prostate cancer is significantly higher than in noncancerous tissues, especially in more aggressive forms of the disease (Gleason score>5). This is in contrast to prostate-specific antigen that is downregulated in higher-grade tumours. The upregulation of MIC-1 in prostate cancer and in advanced and more aggressive prostatic tumours suggests that MIC-1 protein should be evaluated as a potential diagnostic and prognostic biomarker.

Prostate cancer is the most common cancer of North American men. Prostate-specific antigen (PSA), also known as human kallikrein 3 (hK3), according to the approved new nomenclature of the human kallikrein family (Diamandis *et al*, 2000a), is used for early detection and monitoring of prostate cancer (Bilhartz *et al*, 1991; Oesterling, 1991; Diamandis *et al*, 2000b). However, nonmalignant prostatic diseases, especially benign prostatic hyperplasia (BPH) and acute prostatitis, also cause serum PSA elevation, thus complicating the diagnosis of prostatic cancer by PSA measurements alone (Polascik *et al*, 1999). The evaluation of the molecular forms of PSA improves the specificity of PSA (Mitchell *et al*, 2001; Stephan *et al*, 2002). Despite the availability of these tests, there is an urgent need for new biomarkers for early detection of prostate cancer. In accordance with the principles of the development of new biomarkers (Sullivan Pepe *et al*, 2001), one approach would be to search for genes that are overexpressed in prostate cancer.

The macrophage inhibitory cytokine-1 (MIC-1) gene is a member of transforming growth factor-*β* (TGF-*β*) superfamily and was originally isolated from macrophages using the cDNA subtraction method (Bootcov *et al*, 1997). The macrophage inhibitory cytokine-1 gene is also known as growth/differentiation factor-15 (Bottner *et al*, 1999) and placental bone morphogenetic protein (PLAB) (Hromas *et al*, 1997; Thomas *et al*, 2001). Recent reports, using DNA microarray technology, have shown that the MIC-1 gene is more highly expressed in prostate cancer than in BPH tissues (Buckhaults *et al*, 2001; Welsh *et al*, 2001). Until now, there are no quantitative expression data on relatively large groups of patients.

The aim of this study was to investigate the expression of MIC-1 in cancerous and matched noncancerous prostate tissues by quantitative RT–PCR and associate these data with clinicopathological parameters of prostate cancer patients.

## MATERIALS AND METHODS

### Study group

Included in this study were 66 patients who had undergone radical retropubic prostatectomy for prostatic adenocarcinoma at the Charité University Hospital, Berlin, Germany. Patient ages ranged from 48 to 73 years with a mean of 62.7 and a median of 64 years. The patients did not receive any hormonal or other therapy before surgery.

### Prostate cancer tissues

Fresh prostate tissue samples were obtained from the cancerous and noncancerous parts of the same prostates. Small pieces of tissue were gross dissected by an experienced pathologist (GK) immediately after removal of the prostate, snap frozen and stored in liquid nitrogen until analysis, as described previously (Meyer *et al*, 1997). Histological analysis of paraffin-embedded tissue adjacent to these samples was performed by the same pathologist to verify the diagnoses. Only tumour samples that were fully surrounded by malignant tissue according to this analysis were used in this study. We also discarded samples in which benign prostate glands made up more than 10% of the tissue. This way, we minimised the contamination of the tumour sample with benign glands, which is not fully avoidable in prostate cancer unless microdissected tissues were processed. Most of the tumours were located dorsolaterally in the peripheral zone of the prostate. The tissue that we considered as normal was usually taken from the inner zone of the contralateral lobe. Histologically, many of these samples displayed a mild glandular hyperplasia. The criteria of exclusion were prominent inflammatory infiltrates, lack of epithelia due to stromal hyperplasia and prostatic intraepithelial neoplasia. The Ethics Committee of the Charite Hospital approved the use of these tissues for research purposes.

### Total RNA extraction and cDNA synthesis

Tumour tissues were minced with a scalpel, on dry ice, and transferred immediately to 2 ml polypropylene tubes. They were then homogenised and total RNA was extracted using the RNeasy® total RNA isolation system, following the manufacturer's instructions (Qiagen, Valencia, CA, USA). The concentration and purity of RNA were determined spectrophotometrically. Two micrograms of total RNA were reverse-transcribed into first strand cDNA using the Superscript™ preamplification system (Gibco BRL, Gaithersburg, MD, USA). The final volume was 20 *μ*l.

### Quantitative real-time RT–PCR analysis

Two gene-specific primers were designed (MIC-1/F: 5′ CGC GCA ACG GGG ACG ACT 3′ and MIC-1/R: 5′ TGA GC ACC ATG GGA TTG TAG C 3′^11^). Real-time monitoring of PCR reactions was performed using the LightCycler™ system (RocheApplied Science, Indianapolis, IN, USA) and the SYBR green I dye, which binds preferentially to double-stranded DNA. Fluorescence signals, which are proportional to the concentration of the PCR product, are measured at the end of each cycle and displayed on a computer screen (Buckhaults *et al*, 2001). The reaction is characterised by the point during cycling when amplification of PCR products is first detected, rather than the amount of PCR product accumulated after a fixed number of cycles. The higher the starting quantity of the template, the earlier a significant increase in fluorescence is observed (Wittwer *et al*, 1997; Bieche *et al*, 1999). The threshold cycle is defined as the fractional cycle number at which fluorescence passes a fixed threshold above baseline (Bieche *et al*, 1998).

### Endogenous control

For each sample, the amount of the target and of *β* actin, as an endogenous control, was determined using a calibration curve. The amount of the target molecule was then divided by the amount of the endogenous reference, to obtain a normalised target value (Bieche *et al*, 1999).

### Calibration curves

Separate calibration (standard) curves for actin and MIC-1 were constructed using serial dilutions of total cDNA from a healthy human prostate tissue, purchased from Clontech, Palo Alto, CA, USA. The standard curve samples were included in each run. Standards for both MIC-1 and actin RNAs were defined to contain an arbitrary starting concentration, since no primary calibrators exist. Hence, all calculated concentrations are relative to the concentration of the standard.

### PCR amplification

The PCR reaction was carried out on the LightCycler™ system. For each run, a master mixture was prepared on ice, containing 1 *μ*l of cDNA, 2 *μ*l of LC DNA Master SYBR Green I mix, 50 ng of primers and 2.4 *μ*l of 25 mM MgCl_2_. The final volume was adjusted with H_2_O to 20 *μ*l. After the reaction mixture was loaded into a glass capillary tube, the cycling conditions were carried out as follows: initial denaturation at 95°C for 10 min, followed by 42 cycles of denaturation at 95°C for 1 s, annealing at 58°C for 8 s and extension at 72°C for 30 s. The temperature transition rate was set at 20°C s^−1^. Fluorescent product was measured by a single acquisition mode at 92°C after each cycle.

### Melting curve

For distinguishing specific from nonspecific products and primer dimers, a melting curve was obtained after amplification by holding the temperature at 70°C for 30 s followed by a gradual increase in temperature to 99°C at a rate of 0.2°C s^−1^, with the signal acquisition mode set at step, as described. To verify the melting curve results, representative samples of the PCR products were run on 1.5% agarose gels, purified, and cloned into the pCR 2.1-TOPO vector (Invitrogen, Carlsbad, CA, USA) according to the manufacturer's instructions. The inserts were sequenced from both directions using vector-specific primers, with an automated DNA sequencer.

### Statistical analysis

Statistical analysis was performed with SAS software (SAS Institute, Cary, NC, USA). The analyses of differences between MIC-1 expression in noncancerous and cancerous tissues were performed with the nonparametric McNemar test and the Wilcoxon signed ranks test. The binomial distribution was used to compute the significance level of the McNemar test. Relations between different variables were assessed by the Mann–Whitney *U*-test.

## RESULTS

### Expression level of MIC-1 in prostatic tissues

We assessed the quantitative expression of MIC-1 mRNA in the 66 matched pairs of cancerous and noncancerous prostatic tissues. The expression levels of MIC-1 were expressed in arbitrary units, according to a standard curve that was constructed by using serial dilutions of a cDNA obtained from normal prostatic tissue. Results were then normalised by using the ratio of MIC-1/*β*-actin concentration for each sample.

Fifty eight cases showed higher expression level of MIC-1 gene in cancerous prostatic tissues in comparison with noncancerous tissues. Lower expression in cancer was observed in only eight cases. This difference was statistically significant (*P*<0.001) (Table 1

**Table 1:** MIC-1 expression in pairs of noncancerous and cancerous prostatic tissues

**MIC expression**	**Number of patients (%)**	***P*-value**^a^
Higher in cancer *vs* normal	58 (88)	<0.001
Lower in cancer *vs* normal	8 (12)	

a Calculated by McNemar test.

and Figure 1

**Figure 1:**
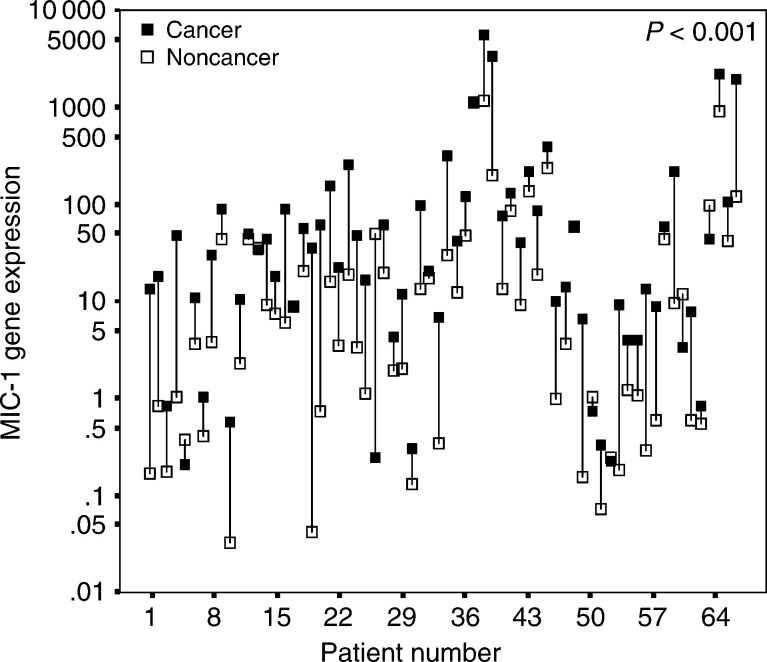
MIC-1 mRNA expression as arbitrary units shown for 66 patients. The black box represents the level in cancerous tissue and the connected white box the respective level of the nonmalignant tissue of the same patient. The *P*-value was calculated by McNemar test.

). The expression levels of MIC-1 gene in cancerous prostatic tissues were significantly higher than that in noncancerous prostatic tissues. Results are summarized in Table 2

**Table 2:** Descriptive statistics for MIC-1 expression (mRNA levels) in noncancerous and cancerous prostatic tissues

	**Mean**^a^	**Standard error^a^**	**Median^a^**	**Range^a^**	***P*-value^b^**
MIC, noncancer (*N*=66)	71	27	6.7	0.03–116	
MIC, cancer (*N*=66)	264	106	32	0.21–5581	<0.001
% Increase^c^	273%	—	375%		

a These values are corrected for actin expression and are unitless ratios.

b Calculated by the Wilcoxon's signed rank test.

c Compared to cancer and assuming that the value in noncancerous tissue is 100%.

and Figure 2

**Figure 2:**
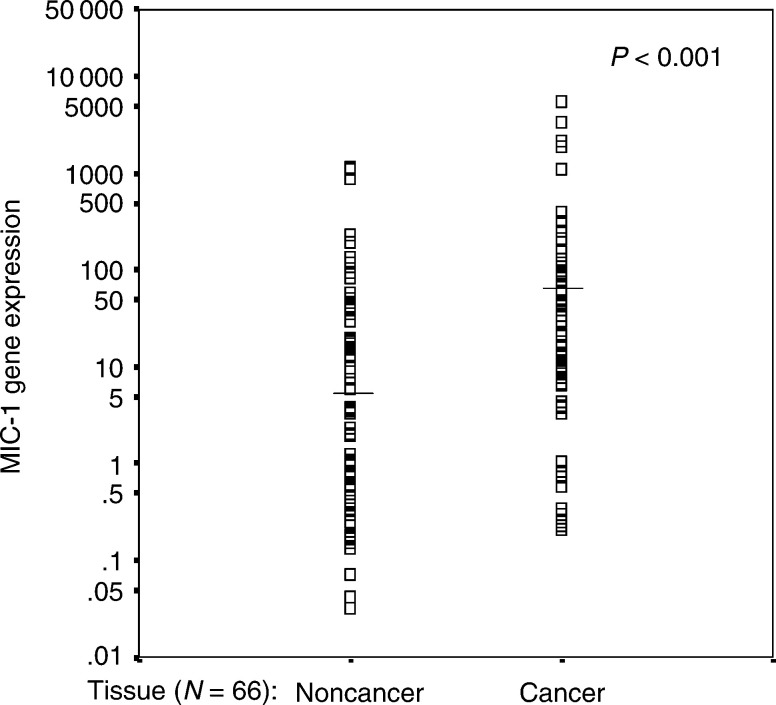
MIC-1 mRNA expression in cancerous and noncancerous prostatic tissues. The horizontal lines indicate the median. The *P*-value was calculated by the Mann–Whitney *U*-test.

. Mean and median values of MIC-1 transcripts were significantly higher in the cancerous tissues by approximately 273–375% (*P*<0.001).

### Association with clinicopathological parameters

The association of MIC-1 mRNA level with clinicopathological parameters in cancerous tissues is shown in Table 3

**Table 3:** MIC expression in cancerous prostatic tissues from 66 patients classified by stage of the disease, Gleason score and tumour grade

	**Total**	**Mean^a^**	**Standard Error^a^**	**Median^a^**	***P*-value^b^**
*Stage*					
I/II	32	145	67	10	0.69
III	34	376	195	37	
					
*Gleason score*					
⩽5	21	117	90	9	0.004
>5	40	373	167	54	
Unknown	5				
					
*Grade*					
G1/2	39	114	50	22	0.86
G3	27	482	246	40	

a These values are corrected for actin expression and are unitless ratios.

b Calculated by the Mann–Whitney *U*-test.

. The expression levels of MIC-1 gene did not show any significant association with tumour stage (*P*=0.69) and tumour grade (*P*=0.86). On the other hand, higher Gleason score (>5 *vs* ⩽5) significantly associated with higher MIC-1 gene expression.

## DISCUSSION

Prostate-specific antigen, also known as hK3, and its molecular forms are the most useful tumour markers for the prostate cancer and hK2, another member of the kallikrein gene family, may help in reducing the number of unnecessary biopsies (Rittenhouse *et al*, 1998). Nevertheless, these serum biomarkers cannot accurately predict the presence of prostate cancer, its aggressiveness or the rate of postoperative PSA failure. New, improved biomarkers might be necessary especially for Gleason 4/5 tumours (Stamey, 2001).

The MIC-1 gene was originally cloned from macrophages using a subtraction-cloning strategy (Bootcov *et al*, 1997). This gene is a member of the TGF-*β* superfamily. Other investigators discovered this gene independently and gave it different names, such as growth/differentiation factor-15 (GDF-15) (Bottner *et al*, 1999) and prostate differentiation factor (PLAB) (Hromas *et al*, 1997; Thomas *et al*, 2001).

Recently, the MIC-1 gene was found to be highly overexpressed in human prostate (Welsh *et al*, 2001) and colorectal cancer (Buckhaults *et al*, 2001) by microarray technology. To confirm these results, and investigate the association with clinicopathological parameters, we assessed the quantitative expression of MIC-1 in a relatively large number of matched prostate cancerous and noncancerous tissues using LightCycler™ technology. Our results showed that MIC-1 gene expression was significantly higher in cancerous prostatic tissue than in noncancerous tissue. Higher Gleason score (>5) cancer expressed significantly more MIC-1 mRNA ( Table 3). These data suggest that MIC-1 gene expression is increased in cancer tissue, compared to normal tissue and its expression is increased when the tumour progresses further. Thus, the level of MIC-1 expression may be a marker of tumour differentiation.

Transforming growth factor-*β* and its receptor were found to be overexpressed in high-grade prostatic intraepithelial neoplasia in the rat ventral prostate. It was reported that high expression of TGF-*β* and its receptors enhance cancer growth and metastasis and are associated with poor prognosis (Wong *et al*, 2000). Preoperative plasma TGF-*β* levels are markedly elevated in men with prostate cancer metastasis and are a strong predictor of biological progression after surgery (Shariat *et al*, 2001). The macrophage migration inhibitory factor (MIF) gene was reported to be elevated in prostate cancer tissues and upregulation of this gene is associated with serum level of MIF (Meyer-Siegler *et al*, 2002).

In conclusion, we report upregulation of the MIC-1 gene in prostate cancer and in advanced and more aggressive prostatic tumours. These data may indicate a possible role for the MIC-1 protein as a future diagnostic and prognostic biomarker. Furthermore, the understanding of the biological function of MIC-1 in prostate may help in delineating its role in prostatic physiology and pathobiology.
